# Toll-Like Receptor 9 Promotes Survival in SERCA2a KO Heart Failure Mice

**DOI:** 10.1155/2017/9450439

**Published:** 2017-04-11

**Authors:** Yangchen Dhondup, Ivar Sjaastad, Øystein Sandanger, Jan Magnus Aronsen, Muhammad Shakil Ahmed, Håvard Attramadal, Alexandra Vanessa Finsen, Lili Zhang, Trine Ranheim, Katrine Alfsnes, Pål Aukrust, Geir Christensen, Arne Yndestad, Leif Erik Vinge

**Affiliations:** ^1^Research Institute of Internal Medicine, Oslo University Hospital, Rikshospitalet, Oslo, Norway; ^2^Center for Heart Failure Research, University of Oslo, Oslo, Norway; ^3^K.G. Jebsen Inflammation Research Center, University of Oslo, Oslo, Norway; ^4^Institute for Experimental Medical Research, Oslo University Hospital, Ullevaal, Oslo, Norway; ^5^Bjørknes College, Oslo, Norway; ^6^Institute for Surgical Research, Oslo University Hospital, Rikshospitalet, Oslo, Norway; ^7^Institute of Clinical Medicine, University of Oslo, Oslo, Norway; ^8^Department of Cardiology, Oslo University Hospital, Rikshospitalet, Oslo, Norway; ^9^Section of Clinical Immunology and Infectious Diseases, Oslo University Hospital, Rikshospitalet, Oslo, Norway; ^10^Department of Internal Medicine, Diakonhjemmet Hospital, Oslo, Norway

## Abstract

*Aim*. Inflammation is important in heart failure (HF). The role of the immune receptor toll-like receptor 9 (TLR9) in HF is not understood and not investigated in diastolic HF. We investigated the role of TLR9 in a murine diastolic HF model caused by cardiomyocyte SERCA2a excision. *Methods and Results*. We crossed SERCA2a KO and TLR9 KO mice to generate four mouse lines. Tamoxifen-induced cardiomyocyte SERCA2a gene excision was carried out in mice, causing diastolic HF. After 7.6 weeks, cardiac functions and dimensions were analyzed by echocardiography and heart tissues were processed. HF mice depleted of TLR9 demonstrated reduced survival compared to SERC2a KO mice, with a median life expectancy of 58 days compared to 63 days. Both HF groups displayed increased left atrium size, lung weight, fetal gene expressions, monocyte/macrophage infiltration, and fibrosis. However, there were no significant differences between the groups. *Conclusion*. In mice with SERCA2a KO-induced diastolic HF, the absence of TLR9 reduced median life expectancy. The cause remains elusive, as all investigated HF parameters were unaltered. Still, these findings support a salutary role of TLR9 in some subsets of HF conditions and underline the importance for future studies on the mechanisms of TLR9 in diastolic HF.

## 1. Introduction

Even with significant clinical improvements in the treatment of HF over the last decades, heart failure (HF) is a growing global challenge with a prevalence of over 23 million worldwide [1, 2]. Moreover, the treatment successes are restricted to HF with reduced ejection fraction (HF-REF). As of today, no HF-specific prognosis-altering intervention can be offered to patients suffering HF with preserved ejection fraction (HF-PEF) [3]. For HF as a whole, it is widely recognized that numerous cardiac molecular signalling systems are pathologically altered. Still, all pharmaceutical treatment strategies as of today target neurohormonal activation. Thus, targeting of signalling systems beyond the neurohormonal axis may be of importance to improve HF treatment.

Immune activation is a key pathogenic mechanism in HF [4–6], and this also includes activation of innate immunity. The innate immune system consists of pattern recognition receptors (PRRs) such as toll-like receptors (TLR) that are activated by evolutionary conserved microbial structures, that is, pathogen-associated molecular patterns (PAMPs). Importantly, PRRs also recognize self-antigens, that is, damage-associated molecular patterns (DAMPs), which are released upon cellular stress or death [7].

TLR9 was first identified as a PRR recognizing cytosine-phosphate-guanine (CpG) repeats within microbial DNA [8], but recent data has demonstrated that endogenous mitochondrial DNA (mtDNA) is a DAMP activating TLR9 [7, 9–11]. The pathophysiological importance of the TLR9 in HF has been addressed experimentally. On the one hand, a study by Oka et al. convincingly demonstrated that increased TLR9 signalling restricted to the cardiomyocyte worsened pressure-overload cardiomyopathy [4]. However, applying the same HF model, Velten et al. demonstrated beneficial properties of systemic TLR9 stimulation [5]. To this end, there are, however, no data on TLR9 activation in diastolic HF. The sarcoplasmic/endoplasmic reticulum Ca^2+^ ATPase (SERCA) is the main cytosolic Ca^2+^ removal transporter and regulates cardiac relaxation [12]. We have previously shown that mice with cardiomyocyte-specific deletion of SERCA2a (SERCA2a KO) develop diastolic HF as compared with wild-type (WT) mice [13, 14]. Using this HF model, we demonstrated that chronic exposure to systemic TLR9 stimulations aggravated diastolic HF [14]. However, systemic TLR9 stimulation caused severe systemic inflammation (including augmented cardiac inflammation) rendering it difficult to conclude whether the worsened cardiac function was a consequence of increased intrinsic cardiac TLR9 stimulation or was an indirect consequence of systemic inflammation.

To further clarify the role of TLR9 in the development of diastolic HF, we examined the effect of TLR9 deficiency in SERCA2a KO-mediated HF.

## 2. Methods

### 2.1. Ethics

All animals were cared for according to the Norwegian Animal Welfare Act, which conforms to the National Institutes of Health guidelines (NIH publication number 85-23, revised 1996). Experiments were approved by the Norwegian National Animal Research Committee (FOTS application 6941). Up to six mice were kept in each cage and housed in a temperature-regulated room with a 12 : 12-hour day-night cycling and had free access to food and water ad libitum.

### 2.2. Generation of Double KO and Induction of Experimental HF

We have previously described the generation of gene-targeted mice with *C57Bl/6J* background. This method allows for temporal control by tamoxifen-induced translocation of Cre from cytosol into nucleus, thus eliciting *α*-myosin heavy chain- (*α*MHC-) dependent and thus cardiac myocyte-specific SERCA2a gene deletion (*α*MHC-MerCreMer-SERCA2a^flox/flox^) [15]. Gene-targeted mice and control mice (*α*MHC-MerCreMer) were generated from the same founder animals and crossed with TLR9^−/−^ mice [8]. The use of TLR9^−/−^ was approved by Professor Shizuo Akira (Department of Host Defense, Osaka University, Japan). Subsequent offsprings were genotyped resulting in four mouse lines: (1) control groups: WT (i.e., *α*MHC-MerCreMer) and TLR9 KO (i.e., *α*MHC-MerCreMer^∗^TLR9^−/−^) and (2) HF groups: SERCA2a KO (i.e., *α*MHC-MerCreMer-SERCA2a^flox/flox^) and SERCA2a/TLR9 KO (i.e., *α*MHC-MerCreMer-SERCA2a^flox/flox^^∗^TLR9^−/−^). Males and females (aged 8–10 weeks) were intraperitoneally (i.p.) injected with a single dose of 100 *μ*l tamoxifen (T5648; Sigma-Aldrich, Oslo, Norway) dissolved in peanut oil to a concentration of 10 mg/ml, inducing nuclear translocation of MerCreMer, but only causing SERCA2a gene deletion in *α*MHC-MerCreMer-SERCA2a^flox/flox^ mice (i.e., SERCA2a KO and SERCA2a/TLR9 KO). Mice were divided into two cohorts: (1) one survival study in which mice were observed daily by an investigator blinded to genotype and intervention, registering morbidity according to prespecified criteria (leading to euthanization by cervical dislocation) and spontaneous death (see Table S1 in Supplementary Material available online at https://doi.org/10.1155/2017/9450439) and (2) a second study where mice were examined by echocardiography 7.6 weeks after induction of HF followed by euthanization and subsequent various investigations on cardiac tissue.

### 2.3. Cardiac Imaging

Echocardiographic examinations were performed with a Vevo 2100 using a 35 MHz linear array transducer (FUJIFILM VisualSonics Inc, Toronto, Canada) on mice anesthetized by a mixture of oxygen and 1.5–1.75% isoflurane and placed in a supine position on a heated pad. Recorded data were analyzed offline using the Vevo 2100 1.1.0 software (VisualSonics). Apart from left ventricle ejection fraction (LVEF), echocardiographic parameters were corrected for tibia length (TL). Relative LV wall thickness was calculated with the formula: [(IVS;d + LVPW;d)/LVID;d]. After recordings and during deep anesthesia (mixture of oxygen and 4-5% isoflurane), mice were euthanized by extraction of the heart. All cardiac imaging was recorded by an investigator blinded to the treatment groups.

### 2.4. Quantification of Cardiac Monocyte/Macrophage Infiltration and Fibrosis

Sections of formalin-fixed and paraffin-embedded heart slices (WT, *n* = 5; TLR9 KO, *n* = 6; SERCA2a KO, *n* = 22; and SERCA2a/TLR9 KO, *n* = 13) were deparaffinized by immersing slides in fresh xylene with subsequent hydration processing followed by 96°C unmasking in citrate buffer (pH 6) for 20 min. Blocking was performed using Rodent block M (Biocare Medical, Concord, CA) followed by one-hour incubation with primary antibody against macrophages (MAC-2; monoclonal rat anti-mouse, clone M3/38, IgG2A dilution 1 : 750; Cedarlane, Burlington, ON, Canada) at room temperature. After washing, slides were incubated for 30 min with peroxidase-conjugated secondary antibody (ImmPRESS Anti-Rat Ig; Vector Laboratories, Burlingame, CA) at room temperature. Sections were developed for 8 min with chromogen for immune peroxidase staining (DAB, Vector Laboratories), before counterstaining with Haematoxylin QS (Vector Laboratories).

Cardiac sections were stained for fibrosis with picrosirius red (WT, *n* = 5; TLR9 KO, *n* = 5; SERCA2a KO, *n* = 22; and SERCA2a/TLR9 KO, *n* = 13) as previously described [16]. For optimal visualization of picrosirius red-positive tissue, haematoxylin staining was excluded. As a measure of total myocardial collagen content, quantitative analysis of tissue contents of hydroxyproline (WT, *n* = 5; TLR9 KO, *n* = 5; SERCA2a KO *n* = 24; and SERCA2a/TLR9 KO, *n* = 13) was performed by HPLC using the AccQ-Fluor reagent kit (Waters Corporation Milford, MA, USA) as previously described [17].

For quantification of MAC-2- and picrosirius red-stained cells and tissue, histological slides were scanned using an automated slide scanner system (Mirax Scan; Carl Zeiss Microscopy, Munich, Germany). Prior to measurement of the stained area, all slides were investigated manually. The whole left ventricle, including septum, was manually evaluated for positive staining. Epicardium and endocardium, as well as artefacts, were manually excluded prior to automated quantification of the stained areas using ImageJ. The stained area was adjusted for the total area of the section, resulting in a relative quantification of the amount of MAC-2-stained cells and picrosirius red staining. Both the operator and analyst were blinded to the different groups during the procedure.

### 2.5. Assessment of Plasma Cytokines and Circulatory Inflammatory Cells

Upon euthanization, arterial blood (approximately 700–1000 *μ*l) was collected at 8 weeks after SERCA2a gene deletion (by a small incision of the carotid artery) into tubes containing 50 *μ*l of 0.5 M EDTA. For measurement of circulating cytokines, plasma was prepared by centrifugation at 2000 × g for 20 min and 4°C and immediately frozen and stored at −80°C. IL-6 and TNF-alpha in mouse plasma were captured with the ProcartaPlex high-sensitivity Luminex assay (custom duoplex; Thermo Fisher Scientific, Waltham, MA) according to manufacturer's protocol and quantified with a Bio-Plex® 200 instrument (Bio-Rad). Plasma levels of interferon-*α* (IFN*α*) and interferon-*β* (IFN*β*) were measured using VeriKine Mouse Interferon Alpha and Beta ELISA Kits (PBL Assay Science, Piscataway, NJ).

Flow cytometry (*n* = 5–9 per group) of circulating blood cells was performed as previously described [18]. In short, blood was drawn as described above. Twenty-four hours later, 100 *μ*l whole blood was blocked using Mouse BD Fc Block (BD Biosciences, San Jose, CA) before labeling with 2.5 *μ*l (0.2 mg/ml) CD11b-APC and Ly6G-PE or 1.0 *μ*l (0.5 mg/ml) CD3e-FITC with subsequent lysis of red blood cells. Flow cytometry analysis was performed blinded to the treatment groups, using a FACSCalibur (BD Biosciences).

### 2.6. RNA Isolation, cDNA Synthesis, and Quantitative RT-PCR (qPCR)

Total RNA (WT, *n* = 5; TLR9 KO, *n* = 6; SERCA2a KO, *n* = 24; and SERCA2a/TLR9 KO, *n* = 15) was isolated from LV myocardial tissue by preprocessing with TRIzol® reagent (Applied Biosystems, Foster City, CA). To ensure optimal RNA quality, subsequent standard isolation using an RNeasy® Mini Kit (Qiagen, Venlo, Netherlands) with DNase treatment of the RNA was performed. All RNA samples were stored at −80°C until further analysis. cDNA was synthesized using the High-Capacity cDNA Reverse Transcription Kit from Applied Biosystems. Target genes were amplified using the Power SYBR® Green Master Mix (Invitrogen Life Technologies Corporation, Carlsbad, CA) and the Applied Biosystems 7900HT Fast Real-Time PCR System. Target gene expression was quantified using the relative standard curve method [19], using a standard curve generated with serial dilution (1 : 5) of a pool of aliquots of sample cDNA, and subsequently normalized to glyceraldehyde 3-phosphate dehydrogenase (GAPDH) gene expression. Primers, designed to span exon-exon boundaries to avoid amplification of genomic DNA, were used for analyzing established parameters of HF and markers of fibrosis. Primer sequences are provided in Table S2.

### 2.7. Statistical Analyses

Unpaired data were analyzed using two-way ANOVA with Tukey's multiple comparison test post hoc with GraphPad Prism 6 (GraphPad, San Diego, CA). Survival analysis was performed using log rank (Mantel-Cox test). Results are shown as mean ± SEM. Probability values of *P* < 0.05 were considered significant.

## 3. Results

### 3.1. Absence of TLR9 Increases Mortality in SERCA2a KO Mice

**Figure 1:**
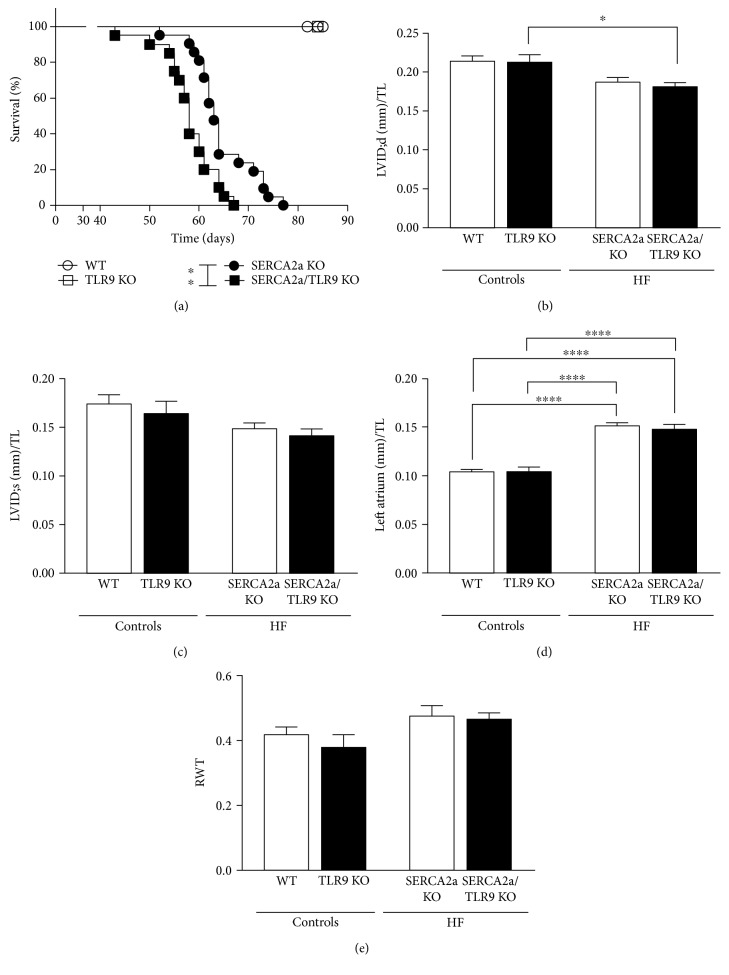
Increased mortality, but no difference in degree of diastolic HF in TLR9-deficient SERCA2A KO mice. (a) Survival analysis: median 58 days in TLR9-deficient SERCA2a KO mice versus 63 days in SERCA2a KO mice. The groups were compared using log rank (Mantel-Cox test: WT, *n* = 7; TLR9 KO, *n* = 3; SERCA2a KO, *n* = 22; and SERCA2a/TLR9 KO, *n* = 20). Of the 52 animals in the survival study, 16 animals were euthanized due to severe illness and 26 died spontaneously. (b) LV inner diastolic diameter/TL (LVID;d, mm). (c) LV inner systolic diameter/TL (LVID;s, mm). (d) Left atrium/TL (mm). (e) Relative wall thickness (RWT). TL, tibia length (mm). The groups were compared using two-way ANOVA with Tukey's multiple comparison test post hoc. Data are shown as mean ± SEM. ^∗^*P* < 0.05, ^∗∗^*P* < 0.01, and ^∗∗∗∗^*P* < 0.0001 versus corresponding control.

All HF animals and none of the control animals reached our prespecified end parameter (death or euthanasia according to prespecified criteria) after injections with tamoxifen in two independent studies. Of the 52 animals in the combined survival study (WT, *n* = 7; TLR9 KO, *n* = 3; SERCA2a KO, *n* = 22; and SERCA2a/TLR9 KO, *n* = 20), 16 animals were euthanized due to objective prespecified criteria of distress (see Table S1) indicating severe HF (SERCA2a KO, *n* = 6; SERCA2a/TLR9 KO, *n* = 10) and 26 animals died spontaneously (SERCA2a KO, *n* = 16; SERCA2a/TLR9 KO, *n* = 10). When including both euthanized and spontaneous deaths, we found reduced life expectancy in SERCA2a KO mice in the absence of TLR9 compared with that in SERCA2a KO mice (median 58 versus 63 days, respectively; *P* = 0.002) (Figure 1(a)). When excluding the euthanized animals, and only comparing spontaneous deaths of SERCA2a KO and SERCA2a/TLR9 KO mice, the outcome remained unchanged (*P* = 0.03). Autopsies of the euthanized mice were conducted and revealed macroscopically enlarged hearts (in particular the atria) and excessive pleural and peritoneal fluid. These observations are clinically very well in agreement with the consequence of severely distorted active relaxation causing increased left ventricular filling pressures with “backward failure” mediating dilatation of the left atrium, increased transpulmonary pressures (causing pleural effusion), and subsequently increased venous pressure (causing ascites/peritoneal effusion).

### 3.2. Depletion of TLR9 Does Not Alter Deteriorated Functional, Structural, or Biochemical Parameters in Hearts of SERCA2a KO Mice

**Figure 2:**
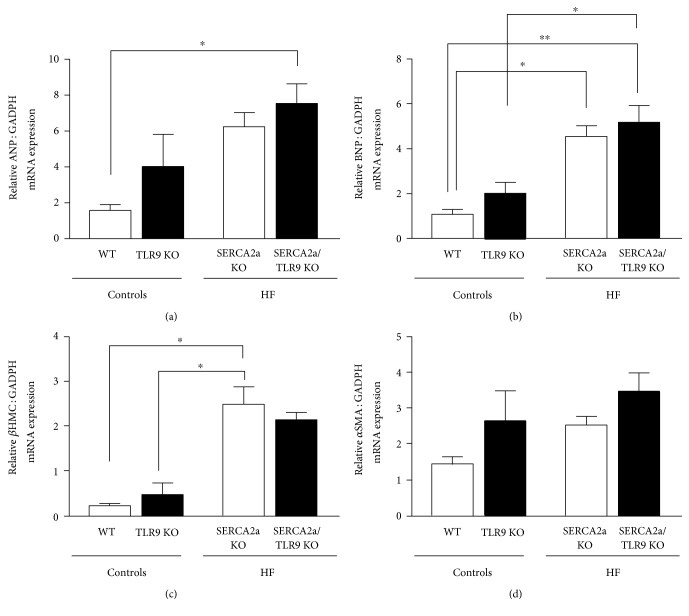
Quantitative RT-PCR on LV myocardial tissue from mice with HF 7.6 weeks after SERCA2a gene excision. (a) ANP, atrial natriuretic peptide, (b) BNP, brain natriuretic peptide, (c) *β*MHC, beta myosin heavy chain, and (d) *α*SMA, alpha smooth muscle actin. The groups (WT, *n* = 5; TLR9 KO, *n* = 6; SERCA2a KO, *n* = 24; and SERCA2a/TLR9 KO, *n* = 15) were compared using two-way ANOVA with Tukey's multiple comparison test post hoc. Data are shown as mean ± SEM. ^∗^*P* < 0.05, ^∗∗^*P* < 0.01 versus corresponding control.

To evaluate the effect of TLR9 on cardiac structure and function, mice were subjected to extensive echocardiographic evaluation 7.6 weeks after induction of SERCA2a excision (Table 1). We have previously shown that SERCA2a KO mice have a reduced LV inner diastolic diameter (LVID;d), subsequently necessitating a lower LV inner systolic diameter (LVID;s) to maintain stroke volume before acute decompensation [14]. This phenotype was seen in both HF groups, with no additional effect of TLR9 deficiency on LV dimensions (Figures 1(b) and 1(c)) nor on left atrium size (Figure 1(d)). A similar pattern was seen for all the other echocardiographic parameters such as LV ejection fraction (LVEF) (Table 1) and relative wall thickness (RWT) (Figure 1(e)), that is, no impact of TLR9 deficiency on the SERCA2a KO phenotype. Also, SERCA2a KO mice showed increased ANP, BNP, and *β*MHC expression in LV tissue, again, with no additional effects of TLR9 deficiency (Figures 2(a), 2(b), and 2(c)). Gene expression of *α*-smooth muscle actin (*α*SMA) was included as a marker of myofibroblast differentiation in the heart. Although *α*SMA tended to be higher in SERCA2a KO mice versus WT (*P* = 0.17) and also to some extent in SERCA2a/TLR9 KO versus SERCA2a KO (*P* = 0.23), these differences did not reach statistical significance (Figure 2(d)).

### 3.3. TLR9 Deficiency Does Not Alter Lung Weight, Inflammation, or Cardiac Fibrosis in SERCA2a KO Mice

**Figure 3:**
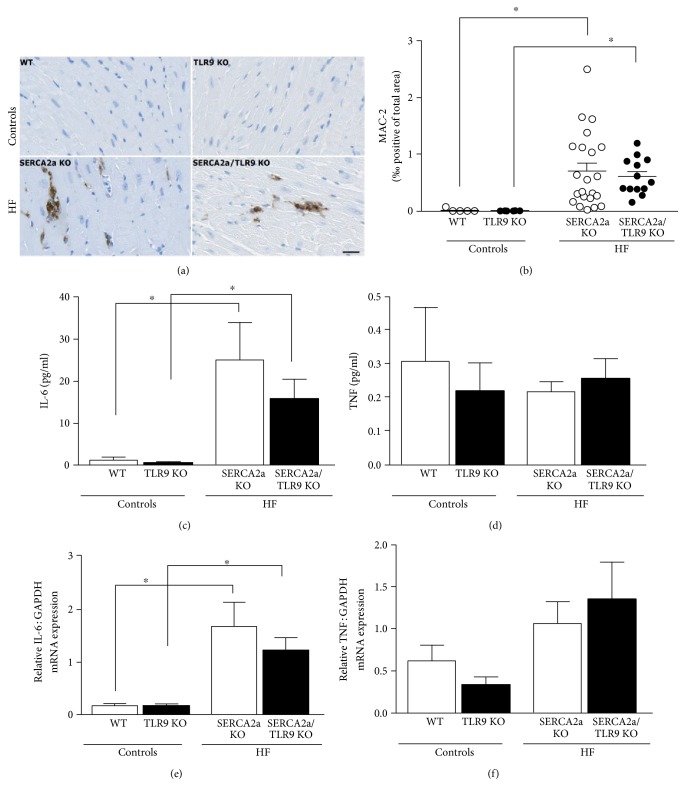
Increased cardiac and systemic inflammation in both HF groups, but no difference between these groups. (a) Representative images of MAC-2-stained cardiac sections. The size marker is 50 *μ*M. (b) Image-based quantification of MAC-2-positive stained cells. (WT, *n* = 5; TLR9 KO, *n* = 6; SERCA2a KO, *n* = 22; and SERCA2a/TLR9 KO, *n* = 13). (c) Plasma levels of interleukin-6 (IL-6) and (d) tumor necrosis factor (TNF) determined by Luminex assays (WT, *n* = 5; TLR9 KO, *n* = 6; SERCA2a KO, *n* = 20; and SERCA2a/TLR9 KO, *n* = 15). (e) Left ventricular gene expression of IL-6 and (f) TNF determined by real-time RT-PCR and presented relative to the gene expression of glyceraldehyde 3-phosphate dehydrogenase (GAPDH) (WT, *n* = 5; TLR9 KO, *n* = 6; SERCA2a KO, *n* = 24; and SERCA2a/TLR9 KO, *n* = 15). The groups were compared using two-way ANOVA with Tukey's multiple comparison test post hoc. Data are mean ± SEM. ^∗^*P* < 0.05 versus corresponding KO control.

**Figure 4:**
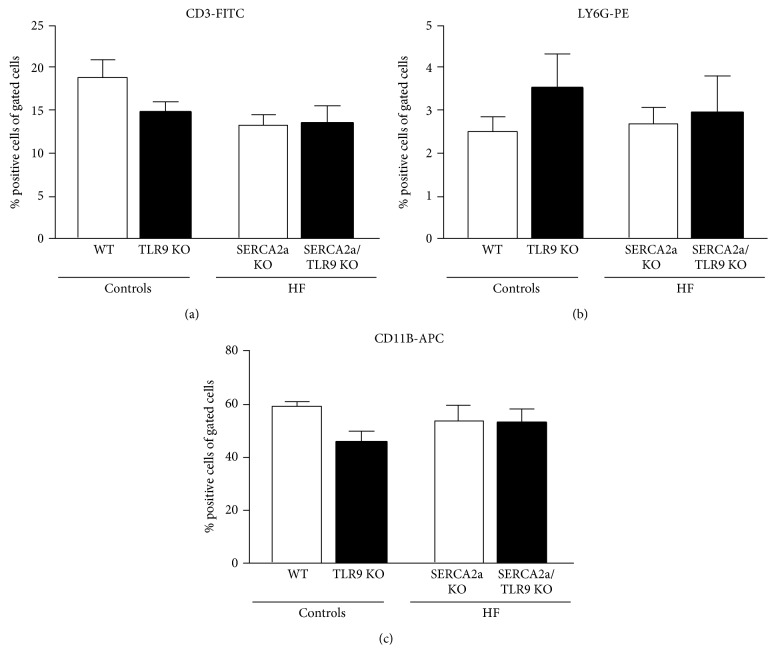
Flow cytometry of CD11b-, CD3-, and LY6G-stained circulating blood cells showed alterations in distribution of cells. (a) CD3-FITC-positive cells, (b) LY6G-PE-positive cells, and (c) CD11b-APC-positive cells. The groups (WT, *n* = 5; TLR9 KO, *n* = 6; SERCA2a KO, *n* = 7; and SERCA2a/TLR9 KO, *n* = 7) were compared using two-way ANOVA with Tukey's multiple comparison test post hoc. Data are mean ± SEM.

Both HF groups displayed increased wet lung weight, supporting a HF phenotype with pulmonary congestion, but with no significant differences between the two HF groups (Table 2). Monocyte/macrophage infiltration was increased in both HF groups compared to control mice, however with no differences between the two HF groups (Figures 3(a) and 3(b)). To further characterize the inflammatory response, we measured plasma levels and myocardial gene expression of IL-6, TNF, IFN*α*, and IFN*β*. In line with the previous results of Vistnes et al. [20], plasma levels of IL-6, but not of TNF, were increased in SERCA2a KO compared to WT (Figures 3(c) and 3(d)). Similarly, we found elevated levels of IL-6 mRNA, but not of TNF mRNA, in hearts from SERCA2a KO mice (Figures 3(e) and 3(f)). The same pattern was found in SERCA2a/TLR9 KO versus TLR9 KO. However, there was no difference, neither in plasma levels nor in cardiac gene expression of IL-6 and TNF, between SERCA2a KO and SERCA2a/TLR9 KO (Figures 3(c) and 3(f)). Plasma levels of both IFN*α* and IFN*β* were not detectable (data not shown). Moreover, cardiac gene expression of IFN*α*_1_ and IFN*β* was very low, with no difference between the four groups (data not shown). For circulating T cells (CD3), granulocytes (Ly6G), and monocytes/macrophages (CD11b), no significant differences were seen between the groups (Figure 4).

**Figure 5:**
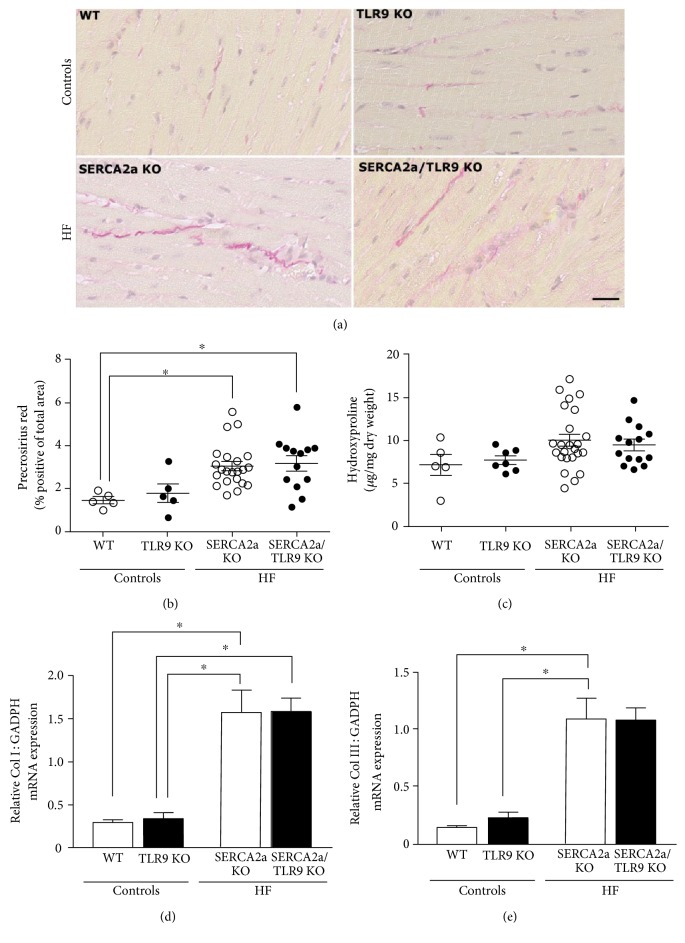
Picrosirius staining and hydroxyproline analysis demonstrated a trend towards increased collagen deposition in both HF groups. (a) Representative picrosirius red-stained cardiac sections. The size marker is 50 *μ*M. (b) Image-based quantification of picrosirius red staining (WT, *n* = 5; TLR9 KO, *n* = 5; SERCA2a KO, *n* = 22; and SERCA2a/TLR9 KO, *n* = 13). (c) Hydroxyproline (*μ*g/ml dry weight) measurements of collagen (WT, *n* = 5; TLR9 KO, *n* = 7; SERCA2a KO, *n* = 24; and SERCA2a/TLR9 KO, *n* = 14). (d, e) Left ventricular gene expression (WT, *n* = 5; TLR9 KO, *n* = 7; SERCA2a KO, *n* = 24; and SERCA2a/TLR9 KO, *n* = 14) of collagen I mRNA (Col I, d) and collagen III mRNA (Col III, e) determined by quantitative RT-PCR and presented relative to expression of GAPDH mRNA. The groups were compared using two-way ANOVA with Tukey's multiple comparison test post hoc. Data are mean ± SEM. ^∗^*P* < 0.05 versus corresponding control.

Diastolic HF involves pathological remodelling of the extracellular matrix in which fibrosis is the most important entity. Analysis of LV myocardial tissue samples by picrosirius red (Figures 5(a) and 5(b)) demonstrated increments of fibrosis in HF, and subsequent measurements of hydroxyproline by HPLC (Figure 5(c)) demonstrated a trend towards the same. In substantiation to these findings, mRNA analyses showed a robust increase in gene-expression levels of both collagen I (Figure 5(d)) and collagen III (Figure 5(e)). However, and in similarity to all other examined functional, structural, biochemical, and inflammatory parameters, we found no significant differences in fibrotic parameters between the two HF groups.

## 4. Discussion

The innate immune system has been suggested as a target for therapy in HF. In the present study, using a model of HF induced by cardiomyocyte-specific deletion of SERCA2a, we found no effect of TLR9 signalling on cardiac remodelling and diastolic function, but still marked a beneficial effect on survival.

The pathophysiological significance of TLR9 in HF has been addressed in some studies [4, 5, 21]. Common to all these studies are that they encompass systolic HF. A clear conclusion as to whether cardiac TLR9 signalling is beneficial or detrimental in HF, however, is not easily drawn from these studies. This may be a consequence of cardiomyocyte-restricted versus systemic TLR9 stimulations [4, 5] and/or chronic versus acute TLR9 stimulations [4, 5]. This discrepancy recently led us to design an additional study employing a different HF model: the murine SERCA2a KO model with mainly a diastolic HF phenotype. When subjecting this model to chronic, systemic TLR9 stimulations, we found increased systemic as well as increased cardiac inflammation and premature death [14]. However, in that study, we could not conclude whether the detrimental effect of TLR9 stimulation was caused by enhanced cardiac TLR9 signalling or was an indirect consequence of elevated systemic inflammation. We, therefore, commenced on performing the current study, generating several comparable gene-targeted mouse models investigating the consequence of endogenous TLR9 in the SERCA2a KO diastolic HF model.

As expected, and demonstrated previously, SERCA2a KO mice displayed clear echocardiographic evidence of HF [13, 15]. These observations were corroborated by findings of increased lung weight. Also, fetal genes were induced and we found explicit evidence of both increased cardiac fibrosis and cardiac and systemic inflammation. Importantly, none of these altered HF parameters differed significantly between SERCA2a KO mice and SERCA2a/TLR9 KO mice nor did circulating levels of monocytes, T cells, and granulocytes. Such findings could easily have led us to conclude that cardiac TLR9 has no pathological significance in HF. However, when observing the natural development of HF in these animals, registering spontaneous death or euthanization due to prespecified criteria of disease/distress, we found that SERCA2a KO mice lacking TLR9 had a lower survival rate compared with SERCA2a KO mice with intact TLR9. It is important to emphasise that an investigator blinded to the genotypes conducted the day-to-day registration of study mice and the results were the same when repeating the experiment. As this is the first study detailing the functional role of *endogenous* TLR9 signalling in HF, future studies employing other models of HF are required to extrapolate this observation towards HF in general. More importantly, however, even though we meticulously conducted a variety of experiments analyzing “classical” alterations in HF, we were not able to provide data suggestive of which mechanism of endogenous cardiac TLR9 signalling conveys its beneficial effects. It may still be that there are subtle differences in the degree of cardiac remodelling and/or inflammation, which this study is underpowered to detect but which are sufficient to explain the rather large difference in hard outcome. Still, given the quite large amount of various experiments yielding nondifferent results, we find this less likely. This leads us to believe that there must be a different cause of mortality (like for instance arrhythmia). Of interest, TLR4 and TLR2 have been suggested to have chronotropic effects on cardiomyocytes [22]. The quest for such a mechanistic explanation must be the basis for future studies. It is, however, intriguing that we also have clinical data suggesting a correlation between high plasma levels of the endogenous TLR9 ligand mtDNA and survival [23].

Our data suggest that while lacking TLR9 may enhance HF in mice with this type of diastolic HF, “over-stimulating” the TLR9 receptor seems to worsen HF development as seen in our previous study and in the study by Oka et al. [4, 14]. Multiple clinical studies have suggested a more refined approach to cardiac inflammation and propose that inflammation may not always be harmful [24, 25]. It may seem that both too much and too little TLR9 activation can provide adverse effects, suggesting that a delicate balance is needed for favourable effects. On this note, it is tempting to suggest a graded stimulation of TLR9 in HF.

## Supplementary Material

To reduce animal suffering and distress, mice were observed daily, registering morbidity according to pre-specified criteria. The individual mice were scored 0 to 4 for different indicators of morbidity as listed in the table. Mice with a total score >8 were euthanized.

## Figures and Tables

**Table 1:** Echocardiographic parameters in SERCA2a KO, SERCA2a/TLR9 KO, and control mice 7.6 weeks after gene excision.

	Controls		HF	
	WT (*n* = 5)	TLR9 KO (*n* = 7)	SERCA2a KO (*n* = 22)	SERCA2a/TLR9 KO (*n* = 14)
LVEF (%)	39.0 ± 5.1	44.9 ± 5.6	42.2 ± 2.6	45.2 ± 4.2
SV (*μ*l)/TL	1.5 ± 0.2	1.7 ± 0.2	1.2 ± 0.1	1.2 ± 0.1^∗^
IVS;d (mm)/TL	0.04 ± 0.002	0.03 ± 0.002	0.04 ± 0.002	0.04 ± 0.002
IVS;s (mm)/TL	0.06 ± 0.002	0.04 ± 0.004	0.05 ± 0.002	0.04 ± 0.003
LVPW;d (mm)/TL	0.05 ± 0.004	0.05 ± 0.006	0.05 ± 0.002	0.05 ± 0.003
LVPW;s (mm)/TL	0.05 ± 0.004	0.06 ± 0.01	0.06 ± 0.003	0.06 ± 0.004
LVvol;d (*μ*l)/TL	3.7 ± 0.3	3.8 ± 0.4	2.9 ± 0.2	2.6 ± 0.2^∗^
LVvol;s (*μ*l)/TL	2.3 ± 0.3	2.2 ± 0.4	1.7 ± 0.2	1.5 ± 0.2

LV: left ventricle; EF: ejection fraction; SV: stroke volume; d: diastolic; s: systolic; IVS: interventricular septum thickness; LVPW: LV posterior wall thickness; LVvol: LV volume; TL: tibia length (mm). The groups were compared using two-way ANOVA with Tukey's multiple comparison test post hoc. Data are mean ± SEM. ^∗^*P* < 0.05 versus corresponding TLR9 KO control.

**Table 2:** Organ weight of SERCA2a KO, SERCA2a/TLR9 KO, and control mice 7.6 weeks after gene excision.

Organ weight (mg/TL)	Controls		HF	
	WT (*n* = 5)	TLR9 KO (*n* = 7)	SERCA2a KO (*n* = 23-24)	SERCA2a/TLR9 KO (*n* = 11–14)
Lung	8.0 ± 0.2	8.3 ± 0.3	10.4 ± 0.3^∗∗^^##^	10.8 ± 0.5^∗∗^^##^
Liver	63.5 ± 6.5	61.6 ± 3.6	58.9 ± 1.9	64.4 ± 2.9
Right ventricle	1.1 ± 0.07	1.1 ± 0.10	1.3 ± 0.04	1.2 ± 0.07
Total heart	5.7 ± 0.2	6.3 ± 0.5	5.7 ± 0.1	5.9 ± 0.2

TL: tibia length (mm). The groups were compared using two-way ANOVA and Tukey's multiple comparison test. Data are mean ± SEM. ^∗∗^*P* < 0.01 versus the WT control and ^##^*P* < 0.01 versus the TLR9 KO control.
