# Supplementary material for: Toll-Like Receptor 9 Promotes Survival in SERCA2a KO Heart Failure Mice
Source: Mediators Inflamm. 2017 Apr 11;2017:9450439. doi: 10.1155/2017/9450439 (PMC5405589; doi:10.1155/2017/9450439)
Supplement: Supplementary file 1 — To reduce animal suffering and distress, mice were observed daily, registering morbidity according to pre-specified criteria. The individual mice were scored 0 to 4 for different indicators of morbidity as listed in the table. Mice with a total score >8 were euthanized. [file 9450439.f1.pdf]

Dhondup et al. " Toll-like receptor 9 promotes survival in SERCA2a KO heart failure mice"

Supplementary Online Material

Supporting Tables

S1 Table. Pre-specified criteria for evaluating morbidity.

Fur appearance	Normal=0	Reduced grooming=1	Untidy fur=2	Very untidy fur=4
Behavior	Normal=0	Reduced activity=1	Very reduced activity=2	
Body weight loss	Normal=0	Loss<5%=1	Loss<10%=2	Loss<15%=3
Movement	Normal=1	Unsteady walk=2	Circulating walk=3	Cannot move=4
Euthanization				If total score is> 8
