# Supplementary material for: Toll-Like Receptor 9 Promotes Survival in SERCA2a KO Heart Failure Mice
Source: Mediators Inflamm. 2017 Apr 11;2017:9450439. doi: 10.1155/2017/9450439 (PMC5405589; doi:10.1155/2017/9450439)
Supplement: Supplementary file 2 [file 9450439.f2.pdf]

S2 Table. Primer sequences used in RT PCR analyses.

Target	Sequence (5'^3')	Acc.nr
ANP	(+)-CAGATCTGATGGATTTCAAGAACCT<br>(-)-GCTTCCTCAGTCTGCTCACTCA	NM_008725
BNP	(+)- GAGCAATTCAAGATGCAGAAGCT (-)-<br>GTGAGGCCTTGGTCCTTCAA	NM_008726
pMHC	(+)-CAACCCTGGAGACCAAGCA<br>(-)-GCTCAAAGCTGTTGAAATCGAA	NM_080728
aSMA	(+)-CGGGAGAAAATGACCCAGATT<br>(-)-GGACAGCACAGCCTGAATAGC	NM_007392
Col I	(+)-CCTGAGTCAGCAGATTGAGAACA<br>(-)-TCGATCCAGTACTCTCCGCTCT	NM_007742
Col III	(+)-TCTATGAATGGTGGTTTTCAGTTCA<br>(-)-TTTTTGCAGTGGTATGTAATGTTCTG	NM_009930
GAPDH	(+)-CCAAGGTCATCCATGACAACTT<br>(-)-AGGGGCCATCCACAGTCTT	NM_008084
IFNa1	(+)-CCTGAACATCTTCACATCAAAGGA<br>(-)-GAGCTGCTGGTGGAGGTCAT	NM_010502
IFNp	(+)-CCATCATGAACAACAGGTGGAT<br>(-)-GAGAGGGCTGTGGTGGAGAA	NM_010510
IL-6	(+)-TCTAATTCATATCTTCAACCAAGAGGTAA<br>(-)-GAATTGGATGGTCTTGGTCCTTA	NM_031168
TNF	(+)-AGACCCTCACACTCAGATCATCTTC<br>(-)-CCACTTGGTGGTTTGCTACGA	NM_013693

Acc.nr.,GenBank Accession number; (+): forward primer, (-): reverse primer. ANP, atrial natriuretic peptide; BNP, brain natriuretic peptide; pMHC, Beta myosin heavy chain; aSMA, alpha smooth muscle actin; Col I, Collagen I; Col III, Collagen III; GAPDH, glyceraldehyde 3-phosphate dehydrogenase; IFN, interferon; IL, interleukin; TNF, tumor necrosis factor.
